# CSF diversion after aneurysmal sub-arachnoid hemorrhage: towards personalized treatment strategies

**DOI:** 10.1186/s13054-025-05788-8

**Published:** 2025-12-02

**Authors:** Julian Klug, Roland Roelz, Giulia Cossu, Nawfel Ben-Hamouda, Stefan Wolf, Urs Pietsch

**Affiliations:** 1 Department of Intensive Care Medicine, Lausanne University Hospital, Lausanne, Switzerland; 2 Stroke Research Group, Department of Clinical Neurosciences, Faculty of Medicine, University Hospital, Geneva, Switzerland; 3 Department of Neurosurgery, Medical Center - University of Freiburg, Faculty of Medicine, University of Freiburg, Freiburg, Germany; 4 Department of Neurosurgery, Lausanne University Hospital, Lausanne, Switzerland; 5 Department of Neurosurgery, Charité-Universitätsmedizin Berlin, Berlin, Germany; 6 Division of Perioperative Intensive Care Medicine, Gallen, Cantonal Hospital St. Gallen, St Switzerland; 7 Department of Emergency Medicine, Inselspital, Bern University Hospital, University of Bern, Bern, Switzerland

**Keywords:** Aneurysmal subarachnoid hemorrhage, Cerebrospinal fluid, Hydrocephalus, External ventricular drain. lumbar drain, Lumbar puncture, Cisternal drain, Delayed cerebral ischemia, Intracranial hypertension

## Abstract

Aneurysmal subarachnoid hemorrhage (aSAH) is a life-threatening condition associated with high morbidity and mortality, with survivors often experiencing long-term neurological and functional deficits. Cerebrospinal fluid (CSF) diversion plays a pivotal role in the acute management of aSAH, both for the treatment of hydrocephalus and for the prevention of delayed cerebral ischemia (DCI) through clearance of blood breakdown products. Four principal modalities are currently employed: lumbar puncture, lumbar, cisternal, and external ventricular drain. Each technique differs in its mechanism of drainage, monitoring capacity, complication profile, and influence on shunt dependency and long-term outcome. High-quality evidence from randomized controlled trials now supports lumbar drainage as the only intervention that significantly reduces the incidence of DCI and has been shown to improve functional outcomes, making it the preferred first-line approach in suitable patients. External ventricular drains remain indispensable in cases of obstructive hydrocephalus or reduced consciousness, while lumbar puncture may be considered in carefully selected low-risk patients. Cisternal drains represent a potential adjunct in those undergoing surgical clipping of the aneurysm. In patients without hydrocephalus, lumbar drains remain the only strategy with demonstrated long-term benefit. Given the heterogeneity of aSAH presentations and the limitations of existing evidence, individualized selection of CSF diversion techniques is warranted. We propose a pragmatic decision-making algorithm to optimize patient outcomes while minimizing iatrogenic complications, which can be adapted to institutional practices and further refined through prospective evaluation.

**Supplementary Information:**

The online version contains supplementary material available at 10.1186/s13054-025-05788-8.

## Background

Aneurysmal subarachnoid hemorrhage (aSAH) results from the rupture of an intracranial aneurysm and subsequent bleeding into the subarachnoid space. It is an uncommon yet highly debilitating condition with substantial global morbidity and case fatality [1, 2]. Survivors frequently experience persistent functional and cognitive impairments that reduce their quality of life and limit their ability to return to work [3]. Key factors influencing functional outcome include early brain injury, hydrocephalus, intracranial hypertension, aneurysm rebleeding, and delayed cerebral ischemia (DCI).

The initial insult following aSAH is a transient global ischemic event precipitated when rising intracranial pressure (ICP) briefly equals arterial pressure [4]. This results in a pronounced sympathetic surge, endothelial damage, and disruption of the blood–brain barrier [5, 6]. Hemoglobin and other blood components remain trapped within the subarachnoid space until erythrocyte membranes degrade and lyse, releasing oxyhemoglobin and other spasmogenic byproducts [7]. Cerebrospinal fluid (CSF) concentrations of oxyhemoglobin and deoxyhemoglobin typically peak around day 7 post-hemorrhage [8]. The progressive accumulation of these factors contributes to the development of DCI, which generally manifests in the same timeframe [6]. It is important to note that vasospasms can occur without any clinical correlation and play only a small part in the complex cascade of vascular and glymphatic dysfunction, inflammation, and spreading depolarizations leading to DCI [9]. To date, nimodipine, a calcium antagonist, is the only medication available to reduce the risk of DCI and recommended to improve long-term functional outcomes [10–12].

Blood breakdown products contribute to the development of acute hydrocephalus in approximately 20% of patients with aSAH [13]. This condition may arise through obstructive mechanisms, such as clot-induced blockage of CSF flow at the aqueduct, malabsorptive mechanisms due to impaired CSF reabsorption by the arachnoid granulations (Pacchionian bodies), or a combination of both [13]. The resulting hydrocephalus can further raise ICP and compromise cerebral perfusion [14].

Drainage of CSF can restore its circulation and reduce ICP. Through the clearance of blood breakdown products and evacuation of clot, diversion of CSF further aims to reduce the incidence of DCI [15]. Maintaining a drain in place offers the added benefits of continuous CSF drainage, ICP monitoring, and the potential for intrathecal therapy; however, it also carries an increased risk of complications, such as infections. Over time, CSF dynamics normalize in most patients, permitting discontinuation of external drainage. However, a subset of patients develop chronic hydrocephalus, necessitating the placement of a ventriculo-peritoneal shunt.

**Fig. 1:**
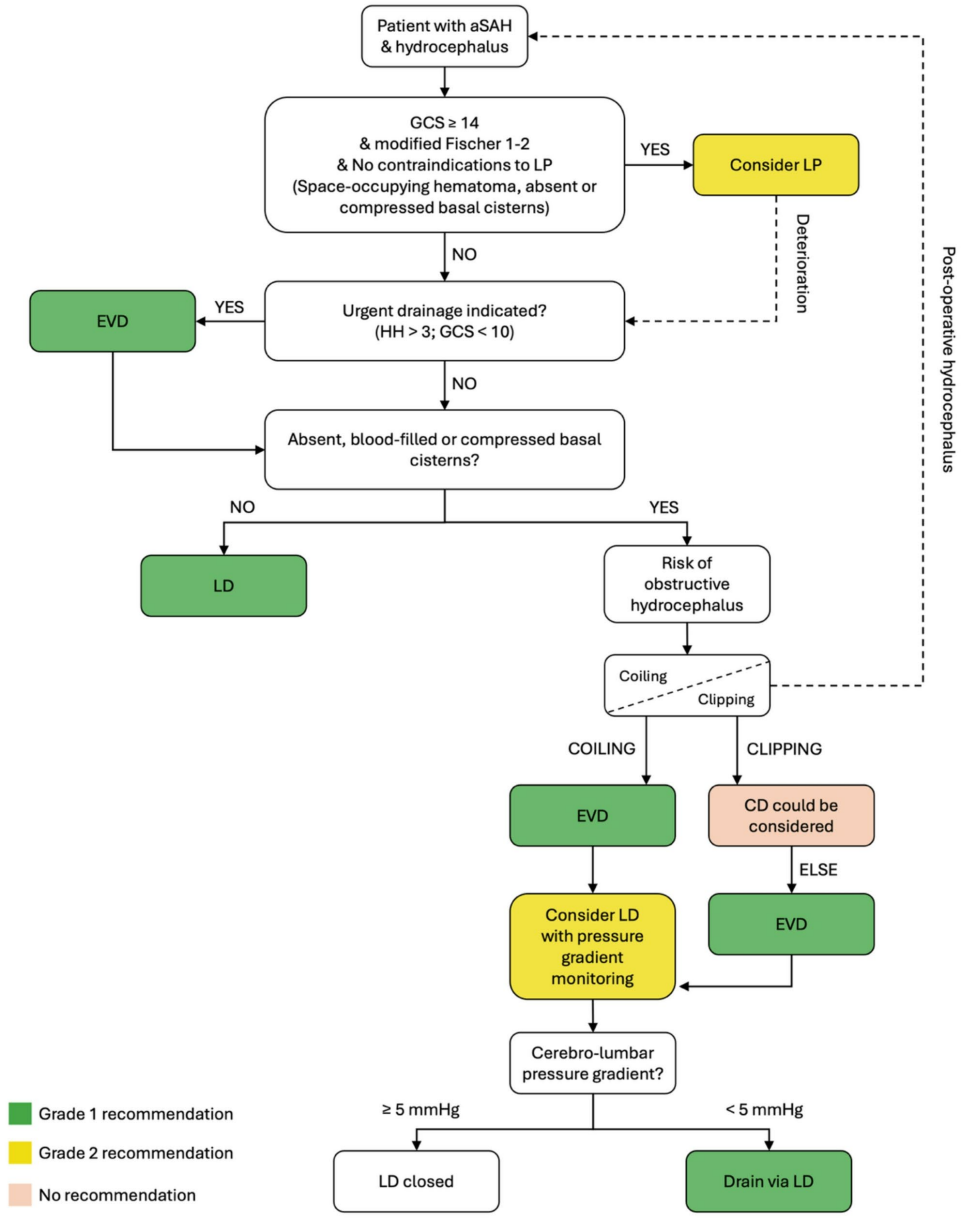
A personalized treatment strategy for CSF diversion after aSAH in patients with acute hydrocephalus. All strategies require correction of existing coagulopathy. The algorithm applies to hydrocephalus developing both before and after treatment of the aneurysm. Grades of recommendation are defined in supplemental Table 2. EVD: external ventricular drain; LD: lumbar drain; CD: cisternal drain; GCS : Glasgow coma scale; HH: Hunt and Hess scale; aSAH : aneurysmal subarachnoid hemorrhage

In the 50 years since the first description of CSF diversion using an external ventricular drain (EVD) for the treatment of acute hydrocephalus following subarachnoid hemorrhage, four main techniques have been developed for this indication [16]. Their differences lie mainly in the CSF space that is being drained, thus influencing the rate, volume, and composition of the fluid being cleared [17]. EVD drain the lateral ventricles, cisternal drains (CD) drain the basal cisterns, lumbar puncture (LP), and lumbar drains (LD) clear the lumbar cistern (Table 1). In recent years, evidence has accumulated reporting the efficacy of each technique in clearing CSF, controlling ICP, preventing DCI, and avoiding complications, as well as their impact on subsequent ventriculo-peritoneal shunt placement. As each CSF diversion technique offers distinct advantages yet differs in the robustness of available evidence, the current approach to CSF management in aSAH requires individualized treatment strategies tailored to each patient’s clinical context. CSF diversion modalities are not mutually exclusive, and some patients may even require multiple sequential or concomitant drainage approaches. In this work, we propose a decision-making algorithm, derived from the current state of the literature and expert opinion, that may be further adapted to institutional practices, with the aim of optimizing long-term outcomes while minimizing iatrogenic complications (Fig. 1). We hope to minimize the risk of repeated, invasive procedures by helping clinicians to choose the right intervention for the right patient at the right time. The evidence and strength of recommendation are graded based on a standard evidence-based medicine grading system (Table 2, Supplemental Tables 1 & 2).

**Table 1:** Advantages, disadvantages and level of evidence behind four CSF drainage techniques

Technique	Eligibility criteria	Advantages	Disadvantages	Level of evidence
Lumbar puncture	Acute hydrocephalus & GCS ≥ 14 & modified Fischer 1–2 & no contraindications (space-occupying hematomas, compressed basal cisterns)	- Minimally invasive - Less VPS compared to EVD - Less complications compared to EVD - Simple and quick to perform - Minimal time and staff requirements	- No ICP monitoring - Single center retrospective data - Never compared to LD - Not evaluated in patients without hydrocephalus - Not evaluated after 72 h	C [20, 23]
Cisternal drain	LD cannot be placed & patient undergoing surgical clipping or decompressive craniectomy	- Less VPS compared to EVD - Access to the basal cisterns for local vasodilator therapy - Clearance of clot in basal cisterns - Possibility to drain large CSF volumes	- Invasive - Performed in OR - Requires specific surgical expertise - Single center retrospective data - Never compared to LD	C [37, 53, 58, 60]
Lumbar drain	All patients with aSAH	- Improves long-term outcomes - Reduces risk of DCI - Less invasive than EVD and CD - Can be combined with EVD & CD - Simple and quick to perform - Minimal time and staff requirements	- Requires concomitant ICP monitoring if GCS < 12 or compressed basal cisterns - Drainage contraindicated if cerebro-lumbar pressure gradient ≥ 5mmHg (with both transducers zeroed at the level of the foramen of Monro)	A [22, 26, 27]
Ventricular drain	Suspicion of raised ICP (Hunt and Hess 4 & 5, GCS < 10) or obstructive hydrocephalus (absent, blood-filled or compressed basal cisterns)	- Best for urgent drainage - First-line for obstructive hydrocephalus - Allows intra-ventricular instillation - Gold standard for ICP monitoring	- Highest rate of infections - Traversal of brain parenchyma - No DCI prophylaxis - Best performed in OR	B [16, 22, 26, 62, 64, 65]

All techniques require correction of existing coagulopathy. Levels of evidence are defined in supplemental Table 1. VPS: ventriculo-peritoneal shunt; ICP: intracranial pressure; EVD: external ventricular drain; LD: lumbar drain; CD: cisternal drain; DCI: delayed cerebral ischemia: OR: operating room

**Table 2:** Comparative studies investigating CSF diversion strategies in aneurysmal subarachnoid hemorrage

Technique	Author and year	Design	Main inclusion & exclusion criteria	Intervention	Outcome	Level of evidence
**Lumbar puncture**	Wenz et al., 2025 [20]	Retrospective, two center	Inclusion: acute hydrocephalus ≤ 72 h Exclusion: diagnostic LP, GCS < 7, space occupying hematoma, complete filling of 3rd or 4th ventricle	LP (± LD and EVD) (*n* = 84) vs. EVD (*n* = 77)	LP was associated with a decreased need for VPS (10% vs. 68%) but no outcome improvement at 3 months	C
	Liu et al., 2025 [23]	Retrospective, single center	Inclusion: all adult aSAH, aneurysm treatment within 48 h Exclusion: therapeutic anticoagulation, pregnancy	Intermittent LP (*n* = 119) vs. LD (*n* = 103)	LP was associated with more unfavorable outcomes at 6 months (42% vs. 21%)	
**Cisternal drain**	Ito et al., 1986 [36]	Retrospective, single center	Inclusion: HH 1–3, clipping within 4 days	CD (*n* = 25) vs. no drain (*n* = 13)	Volume drained by CD was associated with a reduction of DCI	C
	Ogura et al., 1988 [34]	Retrospective, single center	Inclusion: anterior aSAH Exclusion: intraventricular hematoma, vertebrobasilar aneurysm	CD (*n* = 101) vs. no drain (*n* = 31)	CD was associated with a non-significant reduction of DCI in high-risk patients and more hydrocephalus (30% vs. 13%), with an increased need for VPS (13% vs. 7%)	
	Inagawa et al., 1991 [35]	Retrospective, single center	Inclusion: HH 1–4, ≤ 48 h after event, clipping by day 3	CD with irrigation (*n* = 103) vs. no drain (*n* = 37)	CD was associated with a reduction in DCI (15% vs. 30%)	
	Roelz et al., 2017 [58]	Retrospective, single center. matched	Inclusion: HH ≥ 3, modified Fisher ≥ 3, EVD in situ	Stereotactic CD + EVD with irrigation (*n* = 20) vs. standard care (*n* = 60)	CD with irrigation was associated with DCI reduction (15% vs. 42%) and more favorable outcome at discharge (60% vs. 35%)	
	Roelz et al., 2020 [60]	Retrospective, single center	Inclusion: WFNS ≥ 3, modified Fisher ≥ 3, EVD in situ	Stereotactic CD + EVD with irrigation (*n* = 57) vs. standard care (*n* = 214)	CD with irrigation was associated with DCI reduction (8% vs. 18%) and more favorable outcome at 6 months (65% vs. 53%)	
	Garvayo et al., 2023 [37]	Retrospective, single center	Inclusion: acute hydrocephalus Exclusion: death in hospital, lost to follow-up	CD (*n* = 22) vs. EVD (*n* = 67)	CD was associated with a reduced need for VPS (10% vs. 44%) but no outcome improvement at 3 months	
	Roelz et al., 2025 [53]	Retrospective, single center	Inclusion: Hijdra score ≥ 20, aneurysm secured	Catheter irrigation with stereotactic (*n* = 88) or operative CD (*n* = 30), or EVD + LD (*n* = 18) or LD (*n* = 3) vs. standard care (*n* = 543)	Catheter irrigation using mainly CD was associated with DCI reduction (8% vs. 21%) and an improved rate of good outcome in high-risk patients (26% vs. 9%)	
**Lumbar drain**	Al-Tamimi et al., 2012 [26]	RCT, single center	Inclusion: WFNS 1–3, modified Fischer ≥ 2, ≤ 96 h after event	LD (*n* = 105) vs. standard care (*n* = 105) (EVD were used in both groups)	LD was associated with DCI reduction (21% vs. 35%) but no outcome improvement at 6 months.	A
	Borkar et al., 2018 [27]	RCT, single center	Inclusion: HH 2–4, modified Fischer ≥ 2, ≤ 10 d after event Exclusion: suspicion of intracranial hypertension, intracranial mass effect, non-secured aneurysm, meningitis, coiled aneurysms	LD (*n* = 30) vs. standard care (*n* = 30)	LD was associated with DCI reduction (30% vs. 63%) and improved functional outcome at 3 months (median GOS 5 vs. 4)	
	Wolf et al., 2023 [22]	RCT, multicenter	Inclusion: all adult aSAH Exclusion: absent or compressed basal cisterns on admission CT, therapeutic anticoagulation, pregnancy	LD (*n* = 144) vs. standard care (*n* = 143) (EVD were used in both groups)	LD was associated with a reduction in secondary infarctions (29% vs. 40%) and less unfavorable outcomes at 6 months (33% vs. 45%)	
**Ventricular drain**	Kusske et al., 1973 [16]	Retrospective, single center, case-control	Inclusion: hydrocephalus, neurological deterioration, elevated ICP Exclusion: rebleed, intracranial mass	EVD (*n* = 11) vs. no drain (*n* = 9)	EVD was associated with favorable neurological evolution and better survival at 60 d (44% vs. 11%)	B
	Hasan et al., 1989 [62]	Prospective, single center	Inclusion: acute hydrocephalus, ≤ 72 h of event	EVD (*n* = 24) vs. no drain (*n* = 61)	78% of patients improved after EVD, no difference in DCI	
	Ransom et al., 2007 [65]	Retrospective, single center, case-control	Inclusion : HH 4–5	EVD (*n* = 59) vs. no drain (*n* = 19)	No overall outcome difference at 12 months, responders had improved favourable outcomes at 12 months (68% vs. 47%)	

VPS: ventriculo-peritoneal shunt; ICP: intracranial pressure; EVD: external ventricular drain; LD: lumbar drain; CD: cisternal drain; DCI: delayed cerebral ischemia; GCS : Glasgow coma scale; HH: Hunt and Hess scale; WFNS: World Federation of Neurosurgical Societies Scale; aSAH : aneurysmal subarachnoid hemorrhage; RCT: randomised controlled trial

## Lumbar puncture

In the absence of space-occupying hematomas or obstruction of CSF flow, lumbar puncture is the least invasive treatment for acute hydrocephalus. Appropriately selected patients may be treated with a single LP only, during which 25–30 mL of CSF is removed [18, 19]. In case of recurrence, the procedure may be repeated, or an LD inserted. In case of clinical deterioration secondary to the development of acute hydrocephalus, the placement of an EVD is mandated. A recent retrospective study comparing the practice between two leading centers reported a decreased risk of ventriculo-peritoneal shunt dependency (adjusted odds-ratio 0.04 [0.02–0.11]) and complications (adjusted odds-ratio 0.44 [0.21–0.93]), without a significant difference in functional outcome (adjusted odds-ratio 0.59 [0.29–1.20]) when a “LP first” strategy was used [20], compared to a standard care control group. The study excluded patients with hydrocephalus onset after 72 h after aSAH, those with a Glasgow Coma Scale (GCS) < 8, and cases with space-occupying hematoma or complete filling of the third or fourth ventricle.

In a retrospective series, this was an option for 18% of patients with aSAH and acute hydrocephalus, of whom 52% required no further therapy, 29% subsequently received an LD, and 18% an EVD [19]. In patients treated with LP only, a reduced incidence of infections has been demonstrated [21]. Pragmatically, LP represents the simplest, safest, and most expedient intervention. It requires minimal resources and can be performed outside the operating theatre. thereby reducing time-to resolution of hydrocephalus. However, the effect of this treatment strategy on the risk of delayed cerebral ischemia (DCI) and on long-term functional outcomes has not been prospectively evaluated. The intervention was only compared against historical data and never prospectively evaluated against LD, which has later been shown to decrease the incidence of DCI and improve functional outcomes [22]. In an unselected aSAH population, a retrospective single-center study reported an almost doubled risk of unfavorable functional outcome at 6 month with LP, when compared to LD [23]. Consequently, the benefits of this method should be weighed against the risks of neurological injury and DCI. Although the authors of the above-mentioned studies do not detail a threshold for the amount of extravasated blood and report a median Hijdra score of 21 (range 0–42) [15–28], this intervention seems to be mainly an option for patients at low risk of DCI and thus a modified Fisher grade ≤ 2 [24, 25]. Moreover, LP does not allow for continuous monitoring of ICP. While this minimally invasive modality of CSF diversion might be suited for a selected patient population, current evidence remains limited, and most existing data stem from the experience of a single center.

LP for intermittent CSF diversion after aSAH has only been evaluated in small retrospective cohorts (level of evidence C). LP may be considered in select patients with early hydrocephalus and without significant alteration of consciousness (GCS ≥ 14), only small amounts of extravasated blood (modified Fischer 1–2) and without contraindications such as space-occupying hematomas or compressed basal cisterns (grade 2 recommendation) [20]. Outside these criteria, LP is potentially harmful and not recommended [23].

## Lumbar drain

Erythrocytes in cerebrospinal fluid tend to sediment by weight, facilitating their removal in the lumbar region [17]. Three randomized controlled trials with over 500 patients randomized have shown that LD consistently improve outcomes after subarachnoid hemorrhage [22, 26, 27]. The recent EARLYDRAIN trial demonstrated that LD reduced the incidence of secondary infarctions (unadjusted odds-ratio 0.60 [0.37–0.98]) and decreased ICP, but also improved long-term functional outcome (unadjusted odds-ratio 1.67 [1.04–2.70]) with a number-needed-to-treat of 8.3 [22]. The LUMAS trial had previously shown that the risk of DCI was reduced (unadjusted odds-ratio 0.49 [0.26–0.90]) [26]. Of note, there was no difference in ventriculo-peritoneal shunt dependency or infections. No other significant complications were reported and the procedure can be performed safely in the ICU. Around 40% of patients had an EVD placed at the discretion of the treating team. ICP was measured continuously both at cerebral level and using the LD with transducers at the level of the tragus. Intermittent drainage of 5 mL/h through the LD was permitted, provided the cerebro-lumbar gradient (ICP_cerebrum_ - ICP_LD_, with both transducers zeroed at the same level) was beneath 5 mmHg. Although lumbar drainage during episodes of intracranial hypertension was not performed in the randomized controlled trials, a prospective study of patients with refractory ICP and open basal cisterns showed that CSF drainage over an LD lowers ICP safely [28]. A post-hoc analysis of the EARLYDRAIN data has further shown that the total volume of CSF drained over the LD correlates with improved outcomes [29]. However, draining more than 5 mL/h has not yet been formally evaluated and the effect on ventriculo-peritoneal shunt dependency is not known. As LD have been shown to be efficacious for improving outcomes in a broad population of patients with aSAH with and without communicating hydrocephalus, it should now become standard of care [30].

Contraindications to LD are currently debated. A space-occupying hematoma should be considered for surgical evacuation before attempting LD placement. Therapeutic anticoagulation should be balanced prior to any drain implantation. A cerebro-lumbar pressure difference exceeding 5 mmHg, indicating true obstructive hydrocephalus and thus a contraindication, is a rare condition, mostly temporary, and only detectable *after* placement of a lumbar drain [22, 31]. Absent, compressed, or blood-filled basal cisterns on imaging offer only an imperfect assessment of CSF obstruction. In this context, an LD can be placed, kept closed for monitoring, and opened once the gradient has resolved. In patients for whom an EVD is required in the acute phase for rapid control of the ICP, the subsequent placement of an LD allows for more efficient drainage of caudal CSF and facilitates faster EVD weaning [22].

Multiple randomized controlled trials evaluating LD have shown improved long-term functional outcomes (level of evidence A) [22, 26, 27]. Consequently, LD is recommended for most patients after aSAH (grade of recommendation 1). In patients at risk of obstructive hydrocephalus (absent, blood-filled or compressed basal cisterns on imaging), insertion of an LD coupled with an EVD can be beneficial to monitor the cerebro-lumbar pressure gradient (grade of recommendation 2). If the cerebro-lumbar pressure gradient is lower than 5 mmHg, drainage of CSF is recommended (grade of recommendation 1) [22, 31].

## Cisternal drain

The insertion of a cisternal drain (CD) in the basal cisterns, combined with cisternostomy, aims to restore CSF flow at the level of the interpeduncular and prepontine area. This approach is particularly suited for patients undergoing microsurgical aneurysm clipping, as the surgical access to the aneurysm (at least for anterior circulation aneurysms) is generally coupled with the opening of the basal cisterns, making the placement of a CD a straightforward addition to the procedure. Consequently, most studies on cisternal drainage have originated from patient cohorts with surgical aneurysm treatment [32]. The technique entails microsurgical opening of the suprasellar cisterns and the interpeduncular cistern through the membrane of Liliequist [33]. An important distinction must be made regarding the third ventricular access: if fenestration of the lamina terminalis is performed, this constitutes a ventriculo-cisternal drainage system; if not, it represents a purely cisternal drainage approach. The combined technique enables continuous CSF drainage from the basal cisterns as well as the ventricular system and may improve clot washout. Historical series have reported a trend towards a reduction in vasospasm in high grade aSAH, and an association between greater CD drainage volume and both incidence of DCI and mortality [34–36]. Recent retrospective data in patients with surgical aneurysm treatment suggest a decreased risk of ventriculo-peritoneal shunt dependency (unadjusted odds-ratio 0.04 [0.008–0.166]), compared to EVD alone [37]. No differences in functional outcome were reported. By draining both ventricles (if lamina terminalis fenestration is performed) and basal cisterns, CD can drain large CSF volumes, with a mean of 207 mL/day reported in a series of traumatic brain injury patients, allowing efficient control of ICP [38]. However, larger volumes of CSF drainage are associated with an increased risk of ventriculo-peritoneal shunt dependency [34, 39]. CD also allows access to the basal cisterns for the administration of intrathecal vasodilator therapy, fibrinolysis, as well as irrigation [40–54]. Intrathecal spasmolysis has been associated with a reduction in DCI and improved functional outcome in patients with moderate and severe vasospasms in retrospective data from a single center [55]. In patients with a high burden of extravasated blood (Hijdra score ≥ 30), a combination of local fibrinolysis and pressure-guided irrigation was linked to improved functional outcomes in a single-center before-after study [53]. With the increasing adoption of endovascular coiling, alternative access routes for cisternal drainage and therapy have been developed. Initially, lumbar catheters were advanced into the cisterna magna under fluoroscopic guidance [56, 57]. Stereotactic catheter ventriculocisternostomy was introduced to provide a supratentorial access to the basal cisterns independent of the aneurysm securing method [53, 58–60]. A randomized controlled trial evaluating this method is ongoing [61]. Although CD might be promising in selected cases, placement requires specific microsurgical experience or training in stereotaxy. This training gap partly explains the limited adoption to date. Structured training in cisternostomy and stereotactic navigation during neurosurgical residency could address some of these limitations. However, the impact of CD on DCI risk and long-term functional outcomes has never been prospectively assessed, nor has it been compared with a strategy that includes LD. Nonetheless, CD may be suited for patients with contraindications to LD and undergoing surgical aneurysm treatment or decompressive craniectomy, as it offers several of the same theoretical advantages. Current evidence remains however limited and contemporaneous data derives from single institutions. Of note, many contemporary applications of CD combine it with another drain, either EVD or LD, and use fibrinolysis and continuous irrigation to remove the blood from the CSF space.

CD for CSF diversion after aSAH has only been evaluated in small retrospective cohorts using heterogenous techniques (level of evidence C) [37, 53, 58, 60]. In patients in which LD could not be placed, placement of CD during surgical clipping could be considered (no recommendation could be made).

## External ventricular drain

CSF diversion via an EVD is the standard of care for acute hydrocephalus with intracranial hypertension or obstruction of the basal cisterns [10, 62]. The placement of an EVD is a fast, commonly performed, and potentially life-saving procedure recommended as first-line therapy for hydrocephalus and patients with altered consciousness after aSAH [10, 62–65]. While the aggressive evacuation of CSF by continuous external ventricular drainage is theoretically attractive, promising rapid reduction of ICP and accelerated removal of spasmogenic blood breakdown products, current evidence indicates that it does not significantly reduce DCI incidence [29, 66]. Accelerating clot clearance through instillation of intraventricular thrombolytics has been evaluated, but did not result in a reduction in DCI or improvement in functional outcomes in a large randomized controlled trial [67]. Although prophylactic use of intraventricular nimodipine does not improve outcomes [68], retrospective data indicate fewer DCI events when intraventricular nicardipine is used for severe vasospasms [55, 69]. Despite its long history and frequent use, EVD placement remains associated with concerns about complications such as malposition, haemorrhage, and infection, along with substantial variation in the techniques used for insertion and management [70–72]. Notably, EVD traverse brain parenchyma and can cause tract haemorrhages in approximately 15% of patients [73]. Further, EVD carry a higher infectious risk than LD and are thus often performed in the operating theatre [22, 74, 75]. Consequently, intermittent CSF drainage and rapid weaning have been associated with fewer complications and shorter intensive care unit length of stay [66]. Nonetheless, EVD remains the method of choice for aSAH patients who have obstructive hydrocephalus or present with extensive intraventricular hemorrhage. It further remains the gold standard for ICP monitoring [76]. An EVD can be combined with an LD, either with continuous monitoring of the cerebro-lumbar gradient for safe lumbar drainage in patients with a reduced level of consciousness, or, in the absence of obstruction, to allow faster weaning and removal of the EVD, which could further decrease rates of healthcare associated infections [77, 78].

There is limited formal evidence for the use of EVD, which is based on retrospective cohort studies [16, 62, 64, 65], but EVD have been part of the treatment provided in modern randomized controlled trials [22, 26] (level of evidence B). The placement of an EVD is recommended in patients with acute hydrocephalus requiring urgent drainage due to impaired consciousness (Hunt and Hess grades 4 and 5, or a GCS of less than 10) or elevated ICP. It is also recommended in patients at risk of obstructive hydrocephalus, as indicated by absent, blood-filled, or compressed basal cisterns on imaging (grade of recommendation 1) [10].

## Patients without hydrocephalus

In patients without hydrocephalus, CSF diversion aims to prevent delayed cerebral ischemia by clearing spasmogenic blood breakdown products. Although EVD was historically used, ventricular drainage has not consistently demonstrated a reduction in DCI rates, and prophylactic placement in patients without hydrocephalus is not recommended. Taking advantage of the gravity-dependent pooling of subarachnoid blood, drainage from more caudal anatomical sites has shown more consistent results [17, 79]. Trials evaluating LD have actively included patients without hydrocephalus, and LD placement is therefore equally recommended in this patient population. Although the pathophysiological rationale is similar and an increased clot clearance has been demonstrated with serial LP [80], this strategy has not been formally evaluated for the prevention of DCI. Anecdotally, LP has been shown to alleviate headaches in good-grade aSAH patients [81]. CD aim to restore CSF flow and clear breakdown products in the basal cisterns. Although CD drain more caudally than EVD, the prevention of DCI has not been prospectively studied. In patients with contraindications to an LD and undergoing surgical clipping, CD might offer a theoretical advantage over an EVD.

## Conclusion

CSF diversion is now integral to aSAH care, both to treat hydrocephalus and to reduce the risk of DCI. Patients with altered consciousness and acute hydrocephalus are candidates for immediate EVD placement. In patients with low amounts of extravasated blood (modified Fisher < 3), at low risk of DCI, an LP could be considered as a less invasive alternative. LD placement should be considered first-line therapy as it is the only method supported by high-quality evidence for improving long-term outcomes. In patients undergoing surgical clipping, a CD could be considered. However, their use should be subjected to further prospective evaluation before widespread implementation can be recommended. In patients without hydrocephalus, only LD have demonstrated long-term benefit, even though LP and CD share similar pathophysiological mechanisms. Further improvements to actively aid blood clearance, such as ventriculo-lumbar irrigation and CSF filtration are currently under investigation but are yet to be formally evaluated [82]. Finally, the choice of CSF diversion modality must be adapted to the institutional setting and the resources available. Both LP and LD are minimally invasive procedures that require less training than CD and can be performed in the ICU. EVD remain indispensable in emergencies and can be performed in a variety of settings [83, 84].

Given the substantial variability in clinical presentations and the broad range of anatomical characteristics among patients with aSAH, individualized selection of CSF diversion strategies may be beneficial in optimizing patient management. We propose a decision-making algorithm that can be further refined to align with institutional practices, with the objective of optimizing long-term outcomes and minimizing the risk of iatrogenic complications.

## Supplementary Information

Supplementary Material 1.

## Data Availability

No datasets were generated or analysed during the current study.

## Abbreviations

aSAH: aneurysmal subarachnoid hemorrhage
DCI: Delayed cerebral ischemia
ICP: Intracranial pressure
CSF: Cerebrospinal fluid
EVD: External ventricular drain
CD: Cisternal drain
LP: Lumbar puncture
LD: Lumbar drain
GCS: Glasgow coma scale
HH: Hunt and Hess scale
VPS: Ventriculo-peritoneal shunt
OR: Operating room
