# Supplementary material for: CSF diversion after aneurysmal sub-arachnoid hemorrhage: towards personalized treatment strategies
Source: Crit Care. 2025 Dec 2;30:10. doi: 10.1186/s13054-025-05788-8 (PMC12777169; doi:10.1186/s13054-025-05788-8)
Supplement: Supplementary file 1 — Supplementary Material 1. [file 13054_2025_5788_MOESM1_ESM.docx]

Supplementary Material to:

# CSF diversion after aneurysmal sub-arachnoid hemorrhage: towards personalized treatment strategies

Julian Klug^1, 2^, Roland Roelz^3^, Giulia Cossu^4^, Nawfel Ben-Hamouda^1^, Stefan Wolf^5^, Urs Pietsch^6, 7^

1. Department of Intensive Care Medicine, Lausanne University Hospital, Lausanne, Switzerland.
2. Stroke Research Group, Department of Clinical Neurosciences, Faculty of Medicine, University Hospital, Geneva, Switzerland
3. Department of Neurosurgery, Medical Center - University of Freiburg, Faculty of Medicine, University of Freiburg, Freiburg, Germany.
4. Department of Neurosurgery, Lausanne University Hospital, Lausanne, Switzerland.
5. Department of Neurosurgery, Charité-Universitätsmedizin Berlin, Berlin, Germany
6. Division of Perioperative Intensive Care Medicine, Cantonal Hospital St. Gallen, St. Gallen, Switzerland
7. Department of Emergency Medicine, Inselspital, Bern University Hospital, University of Bern, Bern, Switzerland

| **Level of evidence** | **Criteria** |
| --- | --- |
| A | - High-quality evidence from more than 1 RCT - Meta-analyses of high-quality RCTs - One or more RCTs corroborated by high-quality registry studies |
| B | - Moderate-quality evidence from 1 or more RCTs or well-designed, well-executed nonrandomized studies, observational studies, or registry studies - Meta-analyses of such studies |
| C | - Non randomized observational or registry studies with limitations of design or execution - Meta-analyses of such studies - Physiological or mechanistic studies in human subjects |

**Supplemental table 1:** Levels of evidence and corresponding criteria.

| **Grade (strength) of recommendation** | **Criteria** |
| --- | --- |
| 1 | - Benefit vastly greater than risk - Treatment A should be chosen over treatment B |
| 2 | - Benefit probably outweighs risk - Effectiveness is uncertain or not well established |
| No recommendation | - No recommendation can be made. |

**Supplemental table 2:** Grades of recommendation and corresponding criteria.

**References**

1. Wenz F, Tack RWP, Abdulazim A, Van Der Zwan A, Vergouwen MDI, Etminan N, et al. Lumbar Puncture or External Ventricular Drainage as Initial Treatment for Acute Hydrocephalus in Aneurysmal Subarachnoid Hemorrhage—A 2-Center Cohort Study. Neurosurgery. 2025 May 30;

2. Liu J, Chen Q, Sun K, Ding L. Comparative analysis of lumbar cerebrospinal fluid drainage versus lumbar puncture effectiveness in patients with aneurysmal subarachnoid hemorrhage. Sci Rep. 2025 July 1;15(1):21642.

3. Ito U, Tomita H, Yamazaki Sh, Takada Y, Inaba Y. Enhanced cisternal drainage and cerebral vasospasm in early aneurysm surgery. Acta neurochir. 1986 Mar 1;80(1):18–23.

4. Ogura K, Hara M, Tosaki F, Hirai N. Effect of cisternal drainage after early operation for ruptured intracranial aneurysms. Surgical Neurology. 1988 Dec 1;30(6):441–4.

5. Inagawa T, Kamiya K, Matsuda Y. Effect of continuous cisternal drainage on cerebral vasospasm. Acta neurochir. 1991 Mar 1;112(1):28–36.

6. Roelz R, Coenen VA, Scheiwe C, Niesen WD, Egger K, Csok I, et al. Stereotactic Catheter Ventriculocisternostomy for Clearance of Subarachnoid Hemorrhage: A Matched Cohort Study. Stroke. 2017 Oct;48(10):2704–9.

7. Roelz R, Schaefer JH, Scheiwe C, Sajonz B, Csok I, Steiert C, et al. Impact of Stereotactic Ventriculocisternostomy on Delayed Cerebral Infarction and Outcome After Subarachnoid Hemorrhage. Stroke. 2020 Feb;51(2):431–9.

8. Garvayo M, Messerer M, Starnoni D, Puccinelli F, Vandenbulcke A, Daniel RT, et al. The positive impact of cisternostomy with cisternal drainage on delayed hydrocephalus after aneurysmal subarachnoid hemorrhage. Acta Neurochir. 2023 Jan 1;165(1):187–95.

9. Roelz R, Csók I, Overstijns M, Bissolo M, Demerath T, Cimflova P, et al. Decreasing delayed cerebral infarction after aneurysmal subarachnoid hemorrhage using active blood clearance and prevention of delayed cerebral ischemia: results of a 16-year patient registry. J Neurosurg. 2025 June 6;1–11.

10. Al-Tamimi YZ, Bhargava D, Feltbower RG, Hall G, Goddard AJP, Quinn AC, et al. Lumbar drainage of cerebrospinal fluid after aneurysmal subarachnoid hemorrhage: a prospective, randomized, controlled trial (LUMAS). Stroke. 2012 Mar;43(3):677–82.

11. Borkar SA, Singh M, Kale SS, Suri A, Chandra PS, Kumar R, et al. Spinal Cerebrospinal Fluid Drainage for prevention of Vasospasm in Aneurysmal Subarachnoid Hemorrhage: A Prospective, Randomized controlled study. Asian J Neurosurg. 2018;13(2):238–46.

12. Wolf S, Mielke D, Barner C, Malinova V, Kerz T, Wostrack M, et al. Effectiveness of Lumbar Cerebrospinal Fluid Drain Among Patients With Aneurysmal Subarachnoid Hemorrhage: A Randomized Clinical Trial. JAMA Neurology. 2023 Aug 1;80(8):833–42.

13. Kusske JA, Turner PT, Ojemann GA, Harris AB. Ventriculostomy for the treatment of acute hydrocephalus following subarachnoid hemorrhage. J Neurosurg. 1973 May;38(5):591–5.

14. Hasan D, Vermeulen M, Wijdicks EF, Hijdra A, van Gijn J. Management problems in acute hydrocephalus after subarachnoid hemorrhage. Stroke. 1989 June;20(6):747–53.

15. Ransom ER, Mocco J, Komotar RJ, Sahni D, Chang J, Hahn DK, et al. External ventricular drainage response in poor grade aneurysmal subarachnoid hemorrhage: effect on preoperative grading and prognosis. Neurocrit Care. 2007 June 1;6(3):174–80.
